# One-Week Effects of Antibiotic Treatment on Gut Microbiota of Late Neonates With Pneumonia or Meningitis

**DOI:** 10.3389/fped.2021.723617

**Published:** 2021-10-05

**Authors:** Shujing Han, Qiaoru Zhang, Yijun Ding, Ping Chu, Jinjing Zhang, Jin Shi, Shengnan Jia, Caiyun Yang, Jie Lu, Yajuan Wang

**Affiliations:** ^1^Beijing Key Laboratory for Pediatric Diseases of Otolaryngology, Head and Neck Surgery, MOE Key Laboratory of Major Diseases in Children, Beijing Pediatric Research Institute, Beijing Children's Hospital, Capital Medical University, National Center for Children's Health (NCCH), Beijing, China; ^2^Department of Neonatology, Beijing Children's Hospital, Capital Medical University, National Center for Children's Health (NCCH), Beijing, China; ^3^Department of Neonatology, Children's Hospital, Capital Institute of Pediatrics, Beijing, China

**Keywords:** gut microbiota, antibiotic, neonate, pneumonia, meningitis

## Abstract

**Background:** The neonatal period is a critical period for the establishment of the intestinal microbial community. Antibiotics can change the composition of gut microbiota.

**Methods:** Fecal samples were collected from 14 patients with pneumonia and 14 patients with meningitis before and after antibiotic treatment, and fecal samples from five healthy neonates at the 14th and 21st days after birth were collected as well. DNA of fecal samples was extracted, and PCR amplification was performed targeting the V3–V4 variable region of 16S rDNA. After detection by high-throughput sequencing, OTU (operational taxonomic unit) clustering, species annotation, and α diversity analysis were calculated and analyzed statistically.

**Results:** In the healthy control group, the abundance of *Bifidobacterium* increased significantly from 16.75 to 40.42%, becoming the most dominant bacteria. The results of α diversity analysis suggested that the Sobs indexes of the gut microbiota in the pneumonia and meningitis groups were significantly lower than that in the healthy control group (*p* < 0.05). PCoA analysis showed that the gut microbiota of pneumonia and meningitis groups clustered distinctly with the control group (Adonis *p* = 0.001, *R*^2^ = 0.565), and there was no significant change in the diversity of gut microbiota before and after the use of antibiotics.

**Conclusions:** The gut microbiota of neonates with infectious diseases were mainly related to the disease conditions. The initial state of neonatal gut microbiome determines its state after 1-week antibiotic treatment. Antibiotic application with 7 days had little effect on the community richness and some effect on the composition of gut microbiota of neonates with pneumonia or meningitis.

## Introduction

Human gut microbiota refers to the general term of microorganisms inhabiting the human digestive tract system, including bacteria, archaea, viruses, fungi, and protozoa. There are direct or indirect interactions between gut microbiota and the host to form a complex interaction network. This network constitutes a dynamic balance of the micro-ecosystem, which is closely related to human health and disease (1, 2). Current studies show that gut microbiota is closely associated with the occurrence and development of various chronic diseases, including diabetes, hypertension, cardiovascular diseases, brain diseases, and tumors (3). The early formation and development of this system is mainly regulated by human genetics and immunity, and the composition of microbial community is related to the habitat. In addition, diet, lifestyle, and medication (antibiotics) as well as probiotics and pre-biotics also play a key role in regulating microbiota, especially gut microbiota (1).

The infant period is a critical period for the establishment and formation of human normal gut microbiota. The establishment and formation of normal gut microbiota, the development and maturation of the immune system, and the formation of metabolic patterns occur simultaneously (4). During this period, the gut microbiota is in the stage of succession and easily influenced by external factors, especially antibiotics (5). Studies (6, 7) have shown that antibiotics have a great impact on the structure of neonatal intestinal microbial community, and the quick cessation of antibiotic treatment can restore the microbial community. The long-term use of antibiotics in infants could change the normal gut microbiota, further change the body's immune response and metabolic patterns, and has a long-term impact on the human body (8). Although more and more people know that antibiotic treatment can affect the development of neonatal microbiota, the vast majority of previous studies only focused on the use of antibiotics (8), and many studies did not refer to types of diseases suffered by newborns. Thus, the effect of antibiotic use on the gut microbiota of newborns in the treatment of diseases at different infection sites still remains unknown.

In the neonates, pneumonia is the most common infectious disease, while meningitis, especially severe meningitis, is the most severe form of infectious diseases in the neonates. In order to investigate the influences on intestinal flora of neonates by both disease condition and antibiotic treatment, we collected fecal samples of neonates with pneumonia or meningitis, before and after the use of antibiotics in neonatal intensive care unit (NICU). High-throughput sequencing was used to explore the difference of gut microbiota between healthy neonates and neonate patients with infectious diseases (pneumonia or meningitis) and the changes in gut microbiota in patients during the antibiotic treatment.

## Materials and Methods

### Study Design

This prospective case-control study has been approved by the Institutional Review Board of Beijing Children's Hospital, Capital Medical University (Approval No.: 2015-36). Neonates with pneumonia or meningitis in the neonatal ward were included from January 2015 to May 2016. Meanwhile, healthy neonates in the same period were enrolled as the control group. Their general status, the mother's pregnancy, feeding, and other information were collected.

### Patient Inclusion and Exclusion Criteria

All the patients and healthy neonates enrolled were 7- to 28-day-aged, full-term newborns with post-natal exclusive breastfeeding. All the parents of the participating children signed the informed consent form. The neonates in control group were healthy newborns born by the hospital staff in the same period without any antibiotic usage and infectious symptoms.

For diagnostic criteria of pneumonia, please refer to Reference (9). Bacterial pneumonia may be considered if one of the following criteria is met: (1) pulmonary signs: audible phlegm or rales; (2) chest radiograph: diffusely blurred images of the lungs, punctate, and patchy infiltrating images; (3) elevated blood inflammatory indexes (mainly leukocytes, neutrophils, CRP); and (4) bacterial culture of respiratory secretions was positive. Inclusion criteria for patients with pneumonia were (1) full term, >7 days of late neonates. (2) It met the diagnostic criteria of neonatal bacterial pneumonia. (3) Intravenous cefazoxime was used for anti-infection after admission, and the treatment course was 7 days. (4) No antibiotics were used before admission. Exclusion criteria for patients with pneumonia were (1) newborns who were not treated or did not receive regular treatment; (2) newborns with diarrheal diseases and other intestinal diseases, and other infections; (3) newborns who had malformation, congenital, or genetic metabolic diseases; (4) The stool specimen is not qualified.

Purulent meningitis diagnostic criteria were according to Reference (10). Inclusion criteria for patients with meningitis were (1) full term, >7 days of late neonates. (2) meet the diagnostic criteria of neonatal purulent meningitis and meet one of the following criteria: ➀ fever (body temperature ≥38.5°C); ➁ with convulsion, drowsiness, and other nervous system manifestations; ➂ there are circulation instability and other manifestations of infection. (3) Intravenous meropenem was used for anti-infection after admission, and the treatment course was ≥7 days. (4) No antibiotics were used before admission. Exclusion criteria for patients with meningitis were (1) newborns who were not treated or did not receive regular treatment; (2) newborns with diarrhea and other intestinal diseases; (3) newborns who had malformation, congenital or genetic metabolic diseases; (4) The stool specimen is not qualified.

### Specimen Collection

In the patient group, fecal samples were collected at two time points: (1) before the antibiotics were given and (2) the 7th day of antibiotic treatment. Healthy children' fecal samples were collected without urine, dust, and other pollution on their 14th and 21st day after birth. Fecal samples (>1 g) were put in the feces collection boxes, which had been sterilized before use. All samples were stored at −80°C.

### High-Throughput 16S rRNA Amplicon Sequencing

DNA was extracted using a Stool Genomic DNA kit (Beijing ComWin Biotech Co., Beijing, China) following the instructions of the manufacturer. The DNA samples were then sent to Majorbio BioPharm Technology Co. Ltd. (Shanghai, China) for polymerase chain reaction (PCR) amplification, and Illumina high-throughput sequencing. The PCR amplification of the bacterial 16S rRNA gene amplicons (V3 + V4 regions) was performed using specific primers (338F-5′-barcode + ACTCCTACGGGAGGCAGCAG-3′; 806R-5′-GGACTACHVGGGTWTCTAAT-3′). The reaction was carried out with an initial melting step of 95°C for 3 min, followed by 27 cycles of 30 s at 95°C, 30 s at 55°C, and 45 s at 72°C, and a final elongation step of 10 min at 72°C. The 16S rDNA amplification products were electrophoresis in 2% agarose gel, and then detected and recorded on the MultiImager. The AxyPrepDNA gel recovery kit (Axygen, USA) was used to recover the PCR products. The DNA amplicons were used to conduct the high-throughput sequencing on an Illumina MiSeq platform.

### Bioinformatic Analysis

Data analysis was conducted using the i-Sanger platform (http://www.i-sanger.com/) provided by Majorbio Bio-Pharm Technology Co. Ltd. (Shanghai, China). The raw data obtained by sequencing were split into samples according to the joint sequence. Then reads containing more than 10 low-quality reads, reads with joint contamination sequence exceeding 15 bp, and repeated reads were filtered out to obtain high-quality clean data and completed data filtering. Through the overlap relationship between reads, filtered reads were spliced into Tags and clustered into OTUs for species classification with 97% similarity using USEARCH (v7.0.1090). After initial OTU representative sequence was obtained, the chimera generated by PCR amplification was removed from the OTU representative sequence by UCHIME (v4.2.40). Finally, all Tags were compared back to the OTU representative sequence to get the final OTUs representative sequence of each sample by usearch_global.

The OTU representative sequences obtained previously were compared with the database Greengene (V201305) for species annotation using the RDP Classifier (v2.2), and the confidence threshold was set to 0.8. The relative abundance of each sample in phylum, class, order, family, genus, and species was obtained, and the bar plot in the R package was used to obtain the histogram of each sample. Five percent of the species merged into others. Biological diversity analysis was carried out based on the results of OTU analysis, and dilution curves were made according to the analysis results.

### Statistical Analysis

The statistical analysis software SPSS 19.0 was used. Normal distribution of the variables was first tested. For two-group comparison and variables showing normal distribution, Student's *t*-test was used. The *p*-value was two-sided, and *p* < 0.05 was considered statistically significant.

## Results

### General and Clinical Information

**Figure 1:**
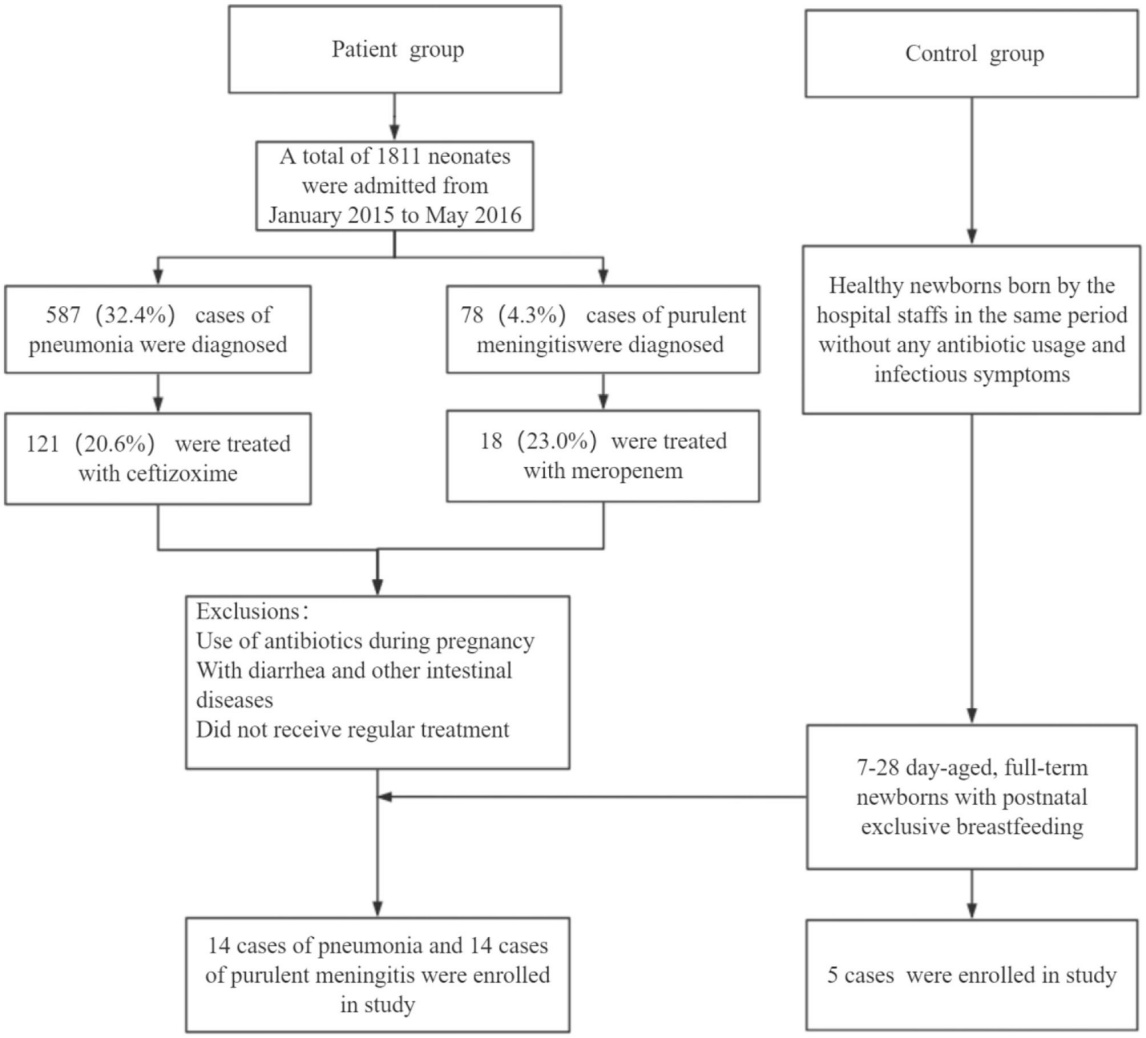
Flow chart of neonate enrollment in our study.

The patients with pneumonia or meningitis were diagnosed according to the diagnosis criteria mentioned above. The selection of antibiotics in the clinical consultation and treatment process is mostly based on clinical experience or pathogenic results. In order to reduce confounding factors, we only chose the pneumonia patients treated by cefazoxime and meningitis patients treated by meropenem. Finally, 14 patients with pneumonia, 14 patients with meningitis, and 5 healthy neonates as control were involved according to our inclusion criteria and exclusion criteria (Figure 1). Table 1 shows the general and clinical examination information of all patients and healthy newborns.

**Table 1:** General information and antibiotic treatment of newborns.

	**Pneumonia** **group (*N* = 14)**	**Meningitis** **group (*N* = 14)**	**Control group** **(*N* = 5)**	***p-*Value**
Male (*n*)	8 (57.1%)	8 (57.1)	3 (60.0)	0.86
Age of admission (days)	16.18 ± 2.53	16.29 ± 1.97	14.00 ± 0.00	0.16
Birth weight (g)	3,415.36 ± 372.01	3,342.14 ± 568.78	3,470.00 ± 349.29	0.69
Gestational age (days)	275.79 ± 6.84	275.57 ± 7.26	274.4 ± 5.18	0.94
1 min Apgar scores ≤ 7	0	0	0	-
Delivery mode				0.10
Cesarean delivery	0	4	0	–
Vaginal delivery	14	10	5	–
Use of antibiotics during pregnancy	0	0	0	–
**Manifestation**				
Fever (≥38.5°C)	11 (78.6%)	14 (100%)	–	
Lung rales	14 (100%)	–	–	
NCPAP	8 (57.1%)	–	–	
Seizure	–	4 (28.6%)	–	
Irritability	–	7 (50%)	–	
Weak response	7 (50%)	11 (78.6%)	–	
Feeding difficulties	7 (50%)	9 (64.3%)	–	
CRT^e^ > 2 s	-	11 (78.6%)	–	
Culture positive	2 (14.3%)	6 (42.9%)	–	
WBC^b^ > 15 × 10^9^/L	2 (14.3%)	3 (21.4%)	–	
CRP > 8 mg/L (n)^a^	5 (35.7%)	5 (35.7%)	–	
NEC^c^ > 60%	2 (14.3%)	5 (35.7%)	–	
Patchy infiltrating images	14 (100%)			
CSF^d^ > 21 × 10^6^/L	–	14 (100%)	–	
CSF protein>1.2 g/L	–	9 (64.3%)	–	
CSF glucose <1.1–2.2 mmol/L	–	7 (50%)	–	
Antibiotic treatment	Ceftizoxime	Meropenem	–	

*^a^C-reactive protein.*

*^b^White blood cell count.*

*^c^Neutrophilic granulocyte percentage.*

*^d^Cerebro-spinal fluid cell count.*

*^e^Capillary refill time*.

### Microbiota Community Structure of Newborns

Through optimization, a total of 2,538,884 high-quality sequences were obtained from 66 samples, with an average length of 447 bp and a length distribution between 420 and 460 bp. The total number of OTU representative sequence was 379. The sparse curve showed that the sequencing data were reasonable (Supplementary Figure 1).

**Figure 2:**
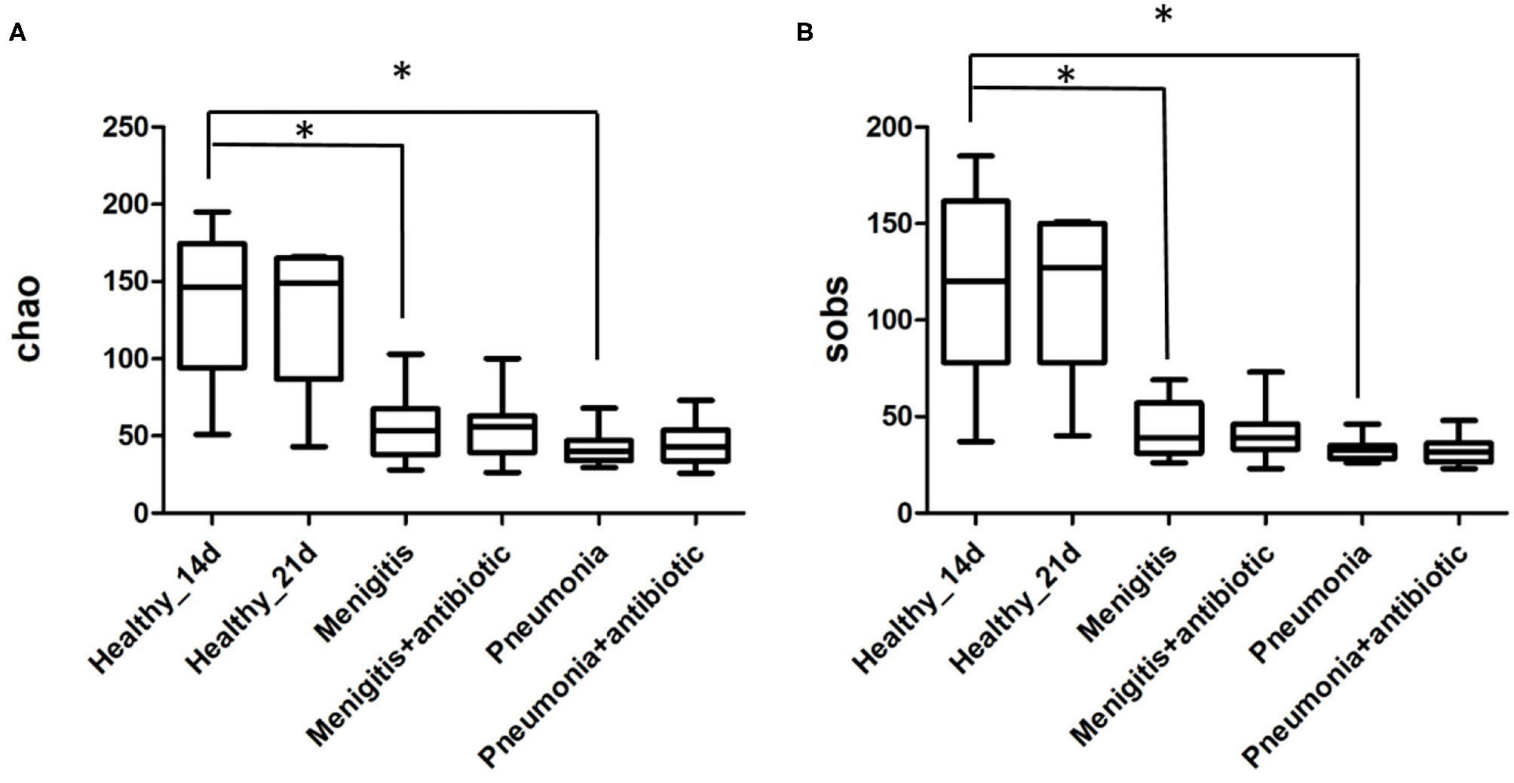
Chao **(A)** and Sobs **(B)** indexes of α diversity analysis. *p < 0.05.

The total number of OTUs in the healthy group (314) was much higher than that in the pneumonia group (138) and meningitis group (172). Alpha-diversity analysis (Figure 2) shows that both the Chao index and the Sobs index in control group were significantly higher than the patient group, indicating that the healthy neonates had a higher community richness and diversity. However, before and after the use of antibiotics, indexes of α-diversity were not significantly changed in both meningitis and pneumonia neonates, which means that bacteria population diversity was not significantly suppressed in individual newborns immediately after antibiotic administration.

**Figure 3:**
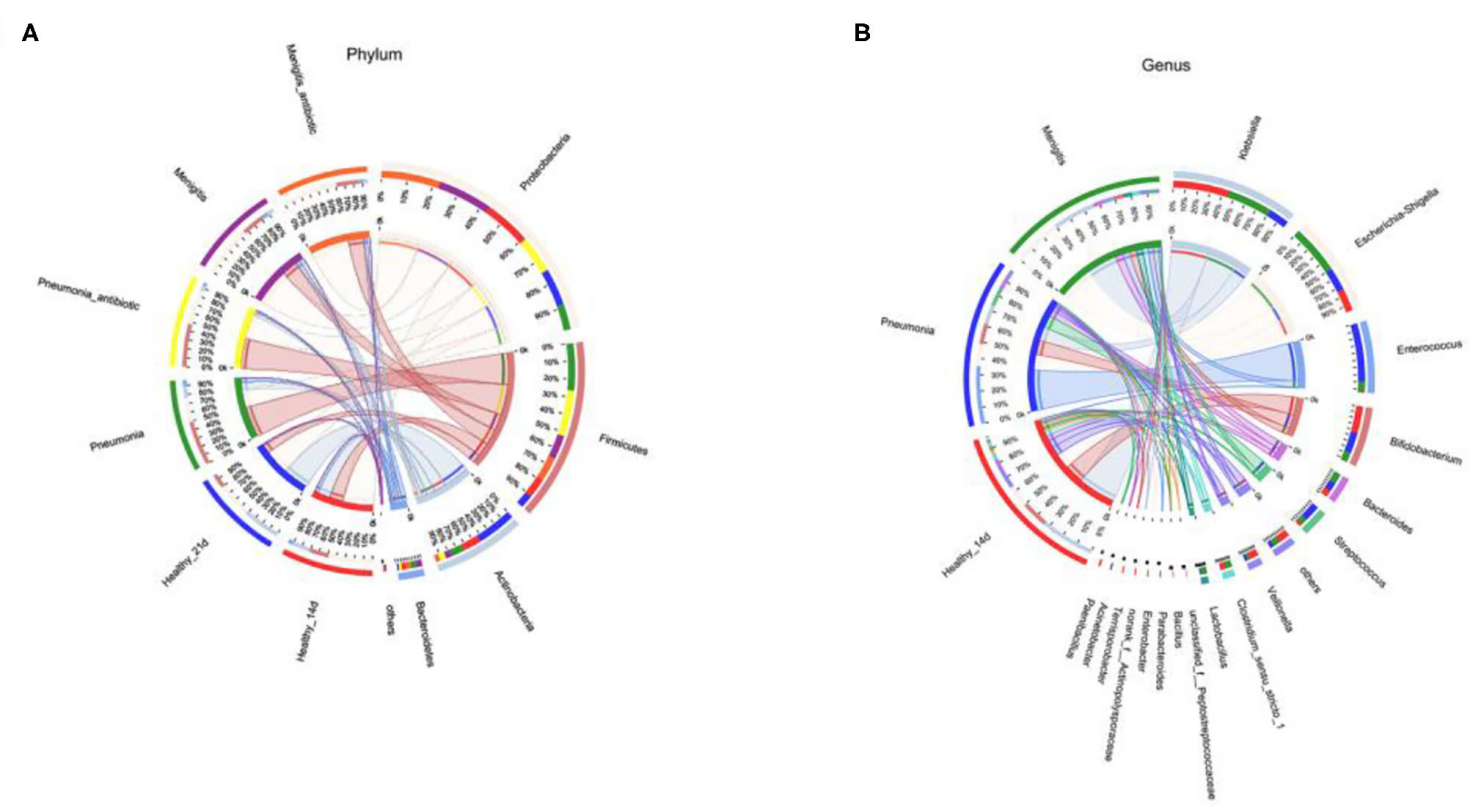
Circos diagram of the relationship between samples at Phylum **(A)** and Genus **(B)** level.

The dominant bacteria of gut microbiota in both healthy and infected newborns were most commonly found in the following four phylum: *Proteobacteria, Firmicutes, Actinobacteria*, and *Bacteroidetes* (Figure 3A). At the genus level (Figure 3B), the dominant bacteria of gut microbiota in our newborns were *Klebsiella, Escherichia–Shigella, Enterococcus*, and *Bifidobacterium*.

### Changes in Gut Microbiota of Healthy Newborns

**Figure 4:**
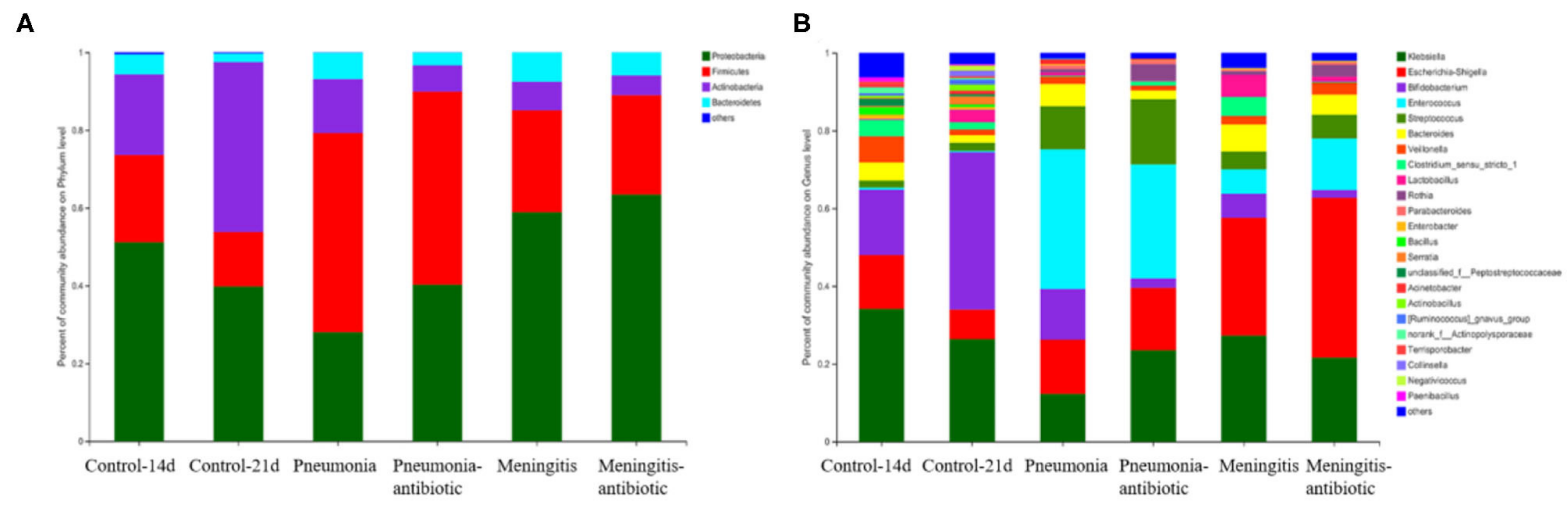
Composition of gut microbiota in phylum **(A)** and genus **(B)** level.

In the healthy control group, we clearly found that with age increase, *Actinobacteria* (from 20.68 to 43.67%) was increasing, while *Proteobacteria* (from 51.17 to 39.78%) and *Firmicutes* (from 22.45 to 14.04%) were decreasing (Figure 4A). At the genus level, the abundance of *Bifidobacterium* (belongs to *Actinobacteria*) increased significantly from 16.75to 40.42%, becoming the most dominant bacteria, and the abundances of *Klebsiella* (belongs to *Proteobacteria*), *Escherichia–Shigella* (belongs to *Proteobacteria*), *Veillonella* (belongs to *Firmicutes*), and *Bacteroides* (belongs to *Proteobacteria*) declined with growth of healthy newborns (Figure 4B). The rise and fall of the genus-level bacteria resulted in changes in the phylum level and also showed normal succession of bacteria in the intestines of healthy newborns.

### Differences in Gut Microbiota Between Healthy Newborns and Patients

**Figure 5:**
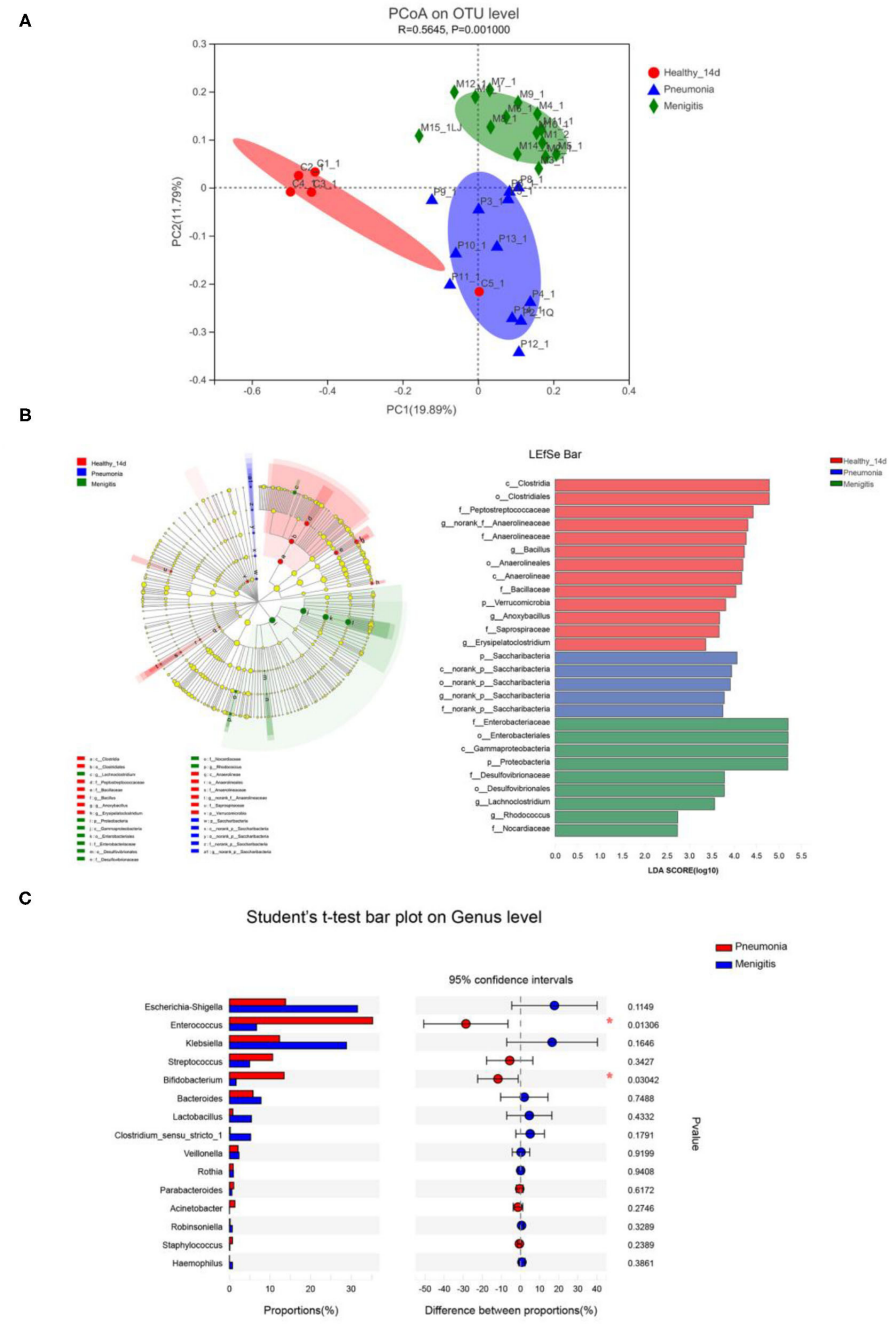
Differences in gut microbiota between healthy newborns and patients before the use of antibiotics. **(A)** Principal coordinate analysis (PCoA) among healthy group and patient groups. **(B)** LEfSe analysis and LDA score among healthy group and patient group. **(C)** Relative abundance of significantly different genera between pneumonia group and meningitis group. *p < 0.05.

We first used principal coordinate analysis (PCoA) on unweighted UniFrac distances to examine the community structures of the gut microbiotas of the three groups. As shown in Figure 5A, the gut microbiota of the pneumonia and meningitis groups clustered distinctly with the control group (Adonis *p* = 0.001, *R*^2^ = 0.565). After 7 days (the patients were treated by antibiotics and the healthy controls did not receive any intervention), the gut microbiota of two groups of patients still clustered distinctly with the control group (Supplementary Figure 2, Adonis *p* = 0.027, *R*^2^ = 0.146).

At the genus level, *Enterococcus* (36.00%), *Escherichia–Shigella* (14.00%), and *Bifidobacterium* (12.93%) were the dominant bacteria in the pneumonia group, and *Escherichia–Shigella* (30.34%), *Klebsiella* (27.28%), and *Enterococcus* (6.34%) were the dominant bacteria in the meningitis group, while *Klebsiella* (34.12%), *Bifidobacterium* (16.75%), and *Escherichia–Shigella* (13.88%) were the dominant bacteria in the control group, but the differences between the groups of the patients and control group were not statistically significant.

LEfSe analysis can be used to analyze the differences in gut microbiota between groups and find out the microbial species that differ between groups, which is conducive to the discovery of biomarkers. As shown in Figure 5B, relative abundance of *Bacillus, Anoxybacillus*, and *Erysipelatoclostridium* was significantly higher in the healthy group than in the meningitis or pneumonia group, while the relative abundance of *Lachnoclostridium* and *Rhodococcus* was significantly higher in the meningitis group, and relative abundance of *Saccharibacteria* was significantly higher in the pneumonia group. The LDA score threshold was 2.

We also compared the gut microbiota of two groups of patients before the use of antibiotics. The α-diversity analysis revealed that the Sobs and Chao index had no significant difference in community richness and diversity between pneumonia and meningitis group (*p* = 0.057, *p* = 0.125). PCoA analysis (Figure 5A) shows that the gut microbiota of pneumonia clustered distinctly with the meningitis groups, indicating that the species composition of gut microbiota from these two groups were quite different. At the genus level (Figure 5C), the abundance of *Enterococcus* (pneumonia 35.32%, meningitis 6.67%) and *Bifidobacterium* (pneumonia 13.49%, meningitis 1.64%) in pneumonia neonates was conspicuously higher than the meningitis groups (*p* = 0.013, *p* = 0.030).

### Difference of Gut Microbiota Before and After the Use of Antibiotics

In the pneumonia group, community richness had no change before and after the use of antibiotics (Figure 2). Meanwhile, the PCoA plot (Supplementary Figure 3) also illustrated the gut microbiota before and after the use of antibiotics were not separated from each other. At the genus level, the abundance of *Bifidobacterium* was lower after 7-day antibiotic treatment (pneumonia 13.49%, pneumonia-antibiotic 2.47%, *p* = 0.057), but the difference was not statistically significant (Supplementary Figure 3).

In the meningitis group, community richness also had no change before and after the use of antibiotics (Figure 2). In addition, the PCoA plot (Supplementary Figure 4) also illustrated the gut microbiota before and after the use of antibiotics were not separated from each other. The abundance of *Bifidobacterium* had no difference before and after the antibiotic treatment (meningitis 1.64%, meningitis-antibiotic 1.94%, *p* = 0.824) (Supplementary Figure 4).

## Discussion

Because the effect of antibiotic use on the gut microbiota of newborns in the treatment of different infectious diseases still remains unknown, we performed this study to investigate the influences on gut microbiota of neonates by disease condition and antibiotic treatment.

To reveal the influences on gut microbiota of neonates by disease condition, we compared the gut microbiota of neonates between the cases (patients with pneumonia or meningitis) and the healthy controls. The results showed that *Bifidobacteria* was the advantage bacterium in the control group, while *Enterococcus* and *Streptococcus* were the advantage bacterium in the pneumonia and meningitis group. The results of α diversity analysis suggested that the Sobs index of the gut microbiota in the pneumonia and meningitis groups was significantly lower than that in the healthy control group (*p* < 0.05). The above data suggested that before the use of antibiotic, the gut microbiota of late neonate with infectious diseases is already quite different from that of healthy neonate. A possible explanation of our result is that the gut microbiota might influence the immunity of the body, and further affect the susceptibility of the neonates to infectious diseases. A study on mice showed (11) that the composition of host intestinal microflora may affect the susceptibility of intestinal pathogens. Zeevi et al. (12) found that the disorder of the gut microbial metabolites could induce the increase production of interleukins and reduce the number of CD4 + CD25 + Foxp3 + cells, which led to the subclinical inflammation. The study of Schirmer et al. (13) expounded the relationship between the difference of gut microbiota and the difference of immune response in healthy individuals and discovered that gut microbiota could interfere with normal immune processes by affecting cytokines. In addition, *Bifidobacterium*, the advantage bacterium in healthy neonates, can prevent pathogenic bacteria from contacting intestinal epithelium, enhance the biological activity of phagocytes of the host, promote the secretion of IgA, enhance cellular immune function, and form intestinal mucosal barrier (14). *Bifidobacteria* can also secrete short-chain fatty acids to reduce the pH value of the intestinal tract and prevent abnormal fermentation of harmful bacteria (15–17).

For humans, antibiotic is a double-edged sword. With the wide application of antibiotics in infectious diseases, antibiotics play an important role in fighting bacterial infection and preventing the spread of pathogenic bacteria in the population and environment. However, the use of antibiotics also has negative effects on human health. Previous studies have shown that antibiotic exposure in childhood can increase the risk of obesity, diabetes, inflammatory bowel disease, asthma, allergy, and other diseases (1). Moreover, antibiotics can directly disrupt gut microbiome and change their composition in both children and adults, leading to a negative impact on the normal gut microbiota (6, 18). This was consistent with our findings. Under normal circumstances, with the increase in daily age, obligate anaerobe *Bifidobacterium* gradually becomes the dominant gut microbiota, but the gut microbiota structure changes under the influence of antibiotics, and the establishment of normal microecological environment in the intestinal tract is delayed, which might affect the colonization of obligate anaerobe and inhibit its growth (14, 19).

In the present study, the species composition of gut microbiota from pneumonia and meningitis groups were quite different, and the abundance of *Enterococcus* and *Bifidobacterium* in pneumonia neonates was conspicuously higher than the meningitis groups (*p* < 0.05), but the composition of gut microbiota had little difference before and after the 7-day antibiotic treatment in patients. Our results revealed that in the initial stage of infectious diseases, the gut microbiota of the patients in these two groups had already been quite different from each other, and the use of antibiotics had little effect on the gut microbiota of the patients, suggesting that the initial state of gut microbiome determined its state after antibiotic treatment. This was consistent with the previous research (20). Raymond et al. (20) exposed 18 young healthy volunteers to a 7-day course of antibiotic, and the results indicated that inter-individual variability at the species level was greater than the effect of the antibiotic. For most participants, it was found that the dominant taxa were not perceptibly affected by the antibiotic.

Our study has some limitations. First, the sample size was small, especially in the healthy control group. Although there were differences in the results of multiple bacteria, and the *p*-values were all high in this study, it may be caused by the small sample size. Second, in addition to antibiotic treatment condition, other extrinsic and intrinsic factors, such as genetic background, pregnancy and childbirth condition, diet of nursing mothers, could also influence the gut microbiota of neonates. Due to the small sample size, we did not consider these influence factors in this study. More verification by expanding the samples is needed to confirm our findings in the future.

## Conclusion

The gut microbiota of neonates with infectious diseases were mainly related to the disease conditions. The initial state of the neonatal gut microbiome determines its state after 1-week antibiotic treatment. Antibiotic application within 7 days had little effect on the community richness and some effect on the composition of gut microbiota of neonates with pneumonia or meningitis.

## Data Availability Statement

The datasets presented in this study can be found in online repositories. The names of the repository/repositories and accession number(s) can be found below: http://db.cngb.org/cnsa/project/CNP0001982/reviewlink/.

## Ethics Statement

The studies involving human participants were reviewed and approved by Institutional Review Board of Beijing Children's Hospital, Capital Medical University (Approval No. 2015-36). Written informed consent to participate in this study was provided by the participants' legal guardian/next of kin. Written informed consent was obtained from the individual(s), and minor(s)' legal guardian/next of kin, for the publication of any potentially identifiable images or data included in this article.

## Author Contributions

JL and YW contributed to conception and design of the study. QZ, YD, JZ, JS, SJ, and CY collected cases and did the experiments. SH and PC performed the statistical analysis. SH and QZ wrote the first draft of the manuscript. All authors contributed to manuscript revision, read, and approved the submitted version.

## Funding

This work was supported by the National Natural Science Foundation of China (81872676), Beijing Natural Science Foundation (7192063), and Special Fund of the Pediatric Medical Coordinated Development Center of Beijing Municipal Administration of Hospitals (XTYB201806).

## Conflict of Interest

The authors declare that the research was conducted in the absence of any commercial or financial relationships that could be construed as a potential conflict of interest.

## Publisher's Note

All claims expressed in this article are solely those of the authors and do not necessarily represent those of their affiliated organizations, or those of the publisher, the editors and the reviewers. Any product that may be evaluated in this article, or claim that may be made by its manufacturer, is not guaranteed or endorsed by the publisher.

## Supplementary Material

Supplementary Figure 1 The sparse curve of healthy group and patient group.

Supplementary Figure 2 PCoA analysis among healthy group and patient groups after 7 days antibiotics treatment in patient groups.

Supplementary Figure 3 Difference of gut microbiota before and after the use of antibiotics in pneumonia group. **(A)** PCoA analysis before and after the use of antibiotics. **(B)** Relative abundance of significantly different genera before and after the use of antibiotics.

Supplementary Figure 4 Difference of gut microbiota before and after the use of antibiotics in meningitis group. **(A)** PCoA analysis before and after the use of antibiotics. **(B)** Relative abundance of significantly different genera before and after the use of antibiotics.
