# Supplementary figures and images for: One-Week Effects of Antibiotic Treatment on Gut Microbiota of Late Neonates With Pneumonia or Meningitis
Source: Front Pediatr. 2021 Oct 5;9:723617. doi: 10.3389/fped.2021.723617 (PMC8525495; doi:10.3389/fped.2021.723617)

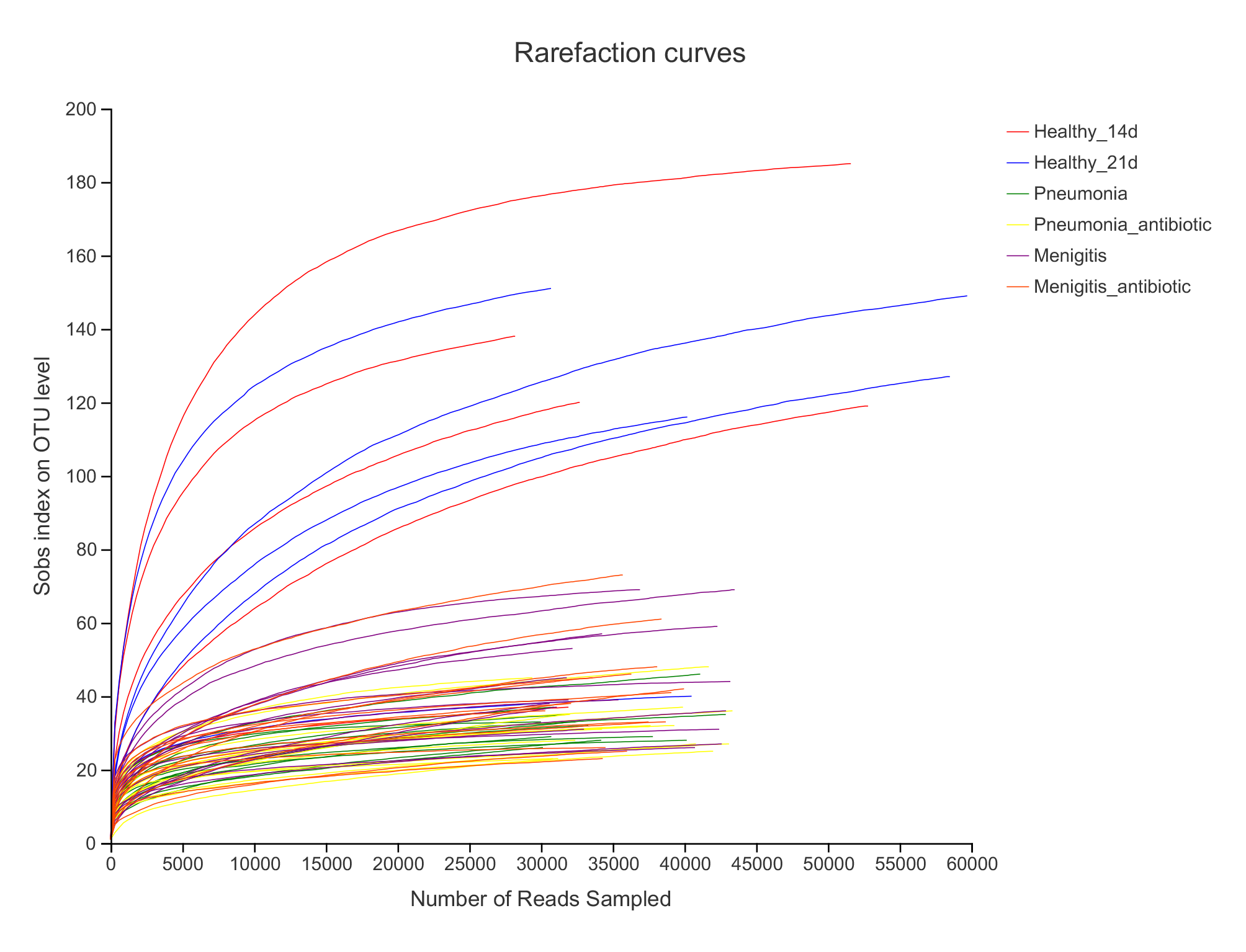

Supplement: Supplementary Figure 1 — The sparse curve of healthy group and patient group. [file Image_1.tif]

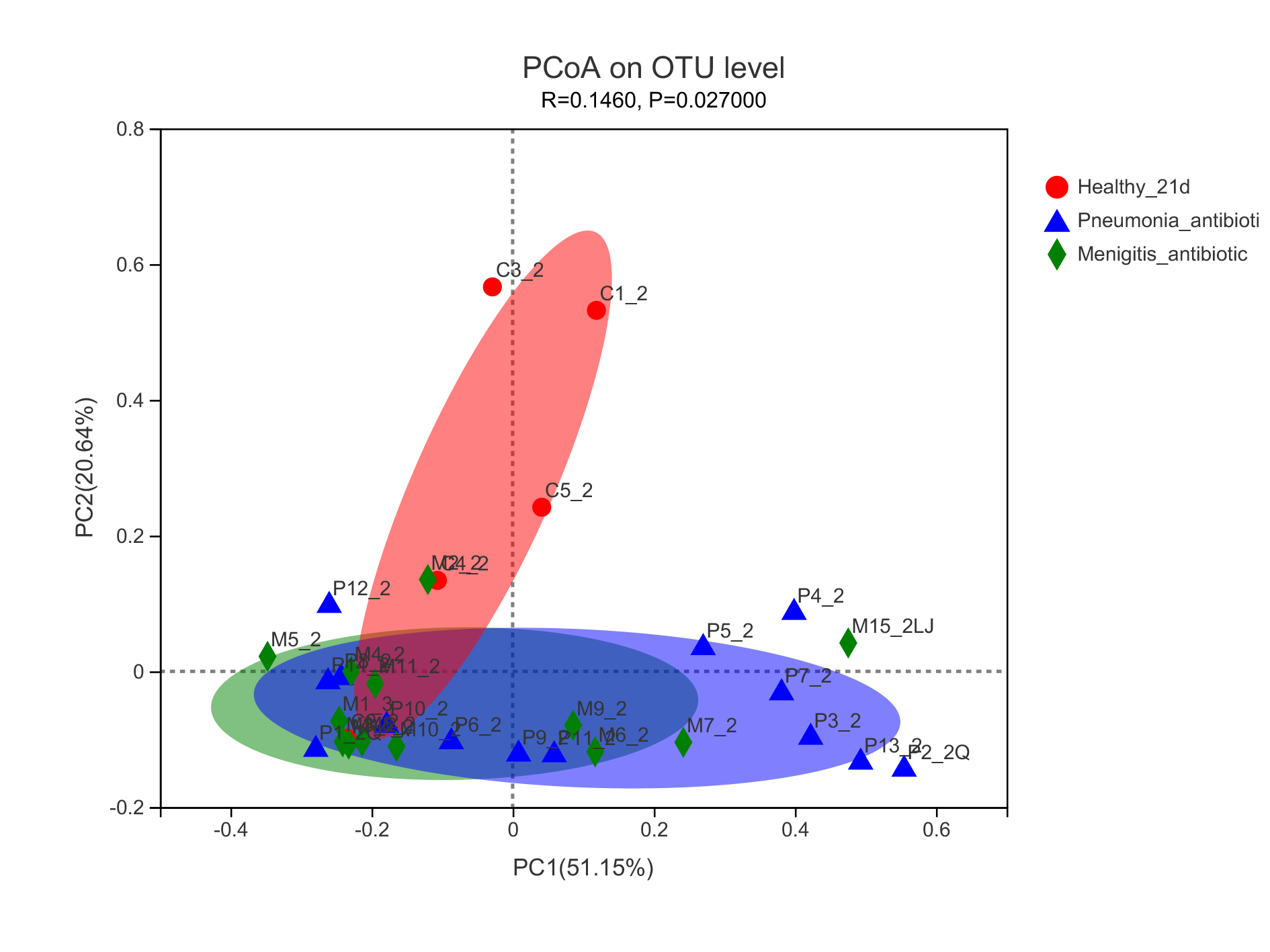

Supplement: Supplementary Figure 2 — PCoA analysis among healthy group and patient groups after 7 days antibiotics treatment in patient groups. [file Image_2.tif]

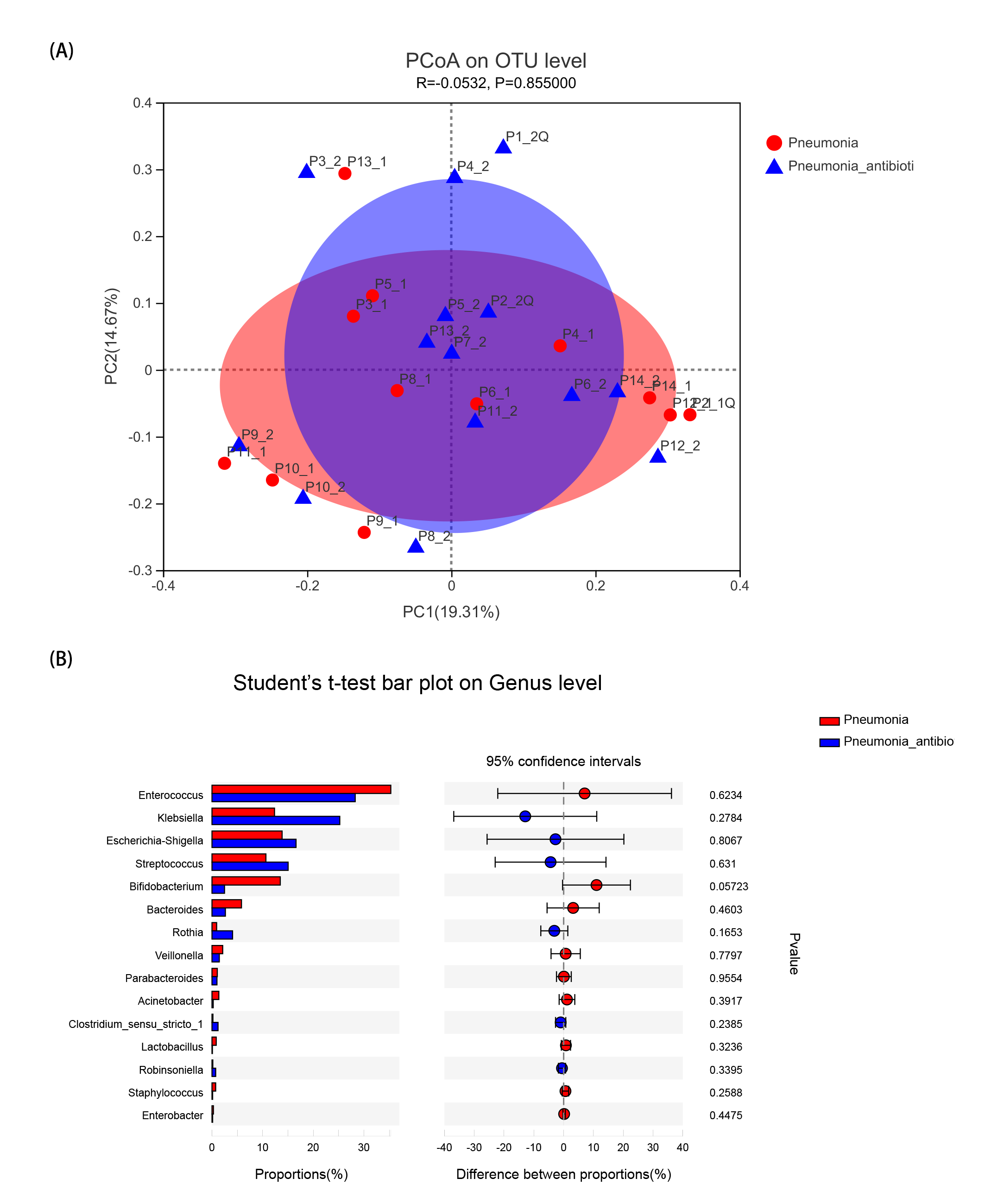

Supplement: Supplementary Figure 3 — Difference of gut microbiota before and after the use of antibiotics in pneumonia group. (A) PCoA analysis before and after the use of antibiotics. (B) Relative abundance of significantly different genera before and after the use of antibiotics. [file Image_3.tif]

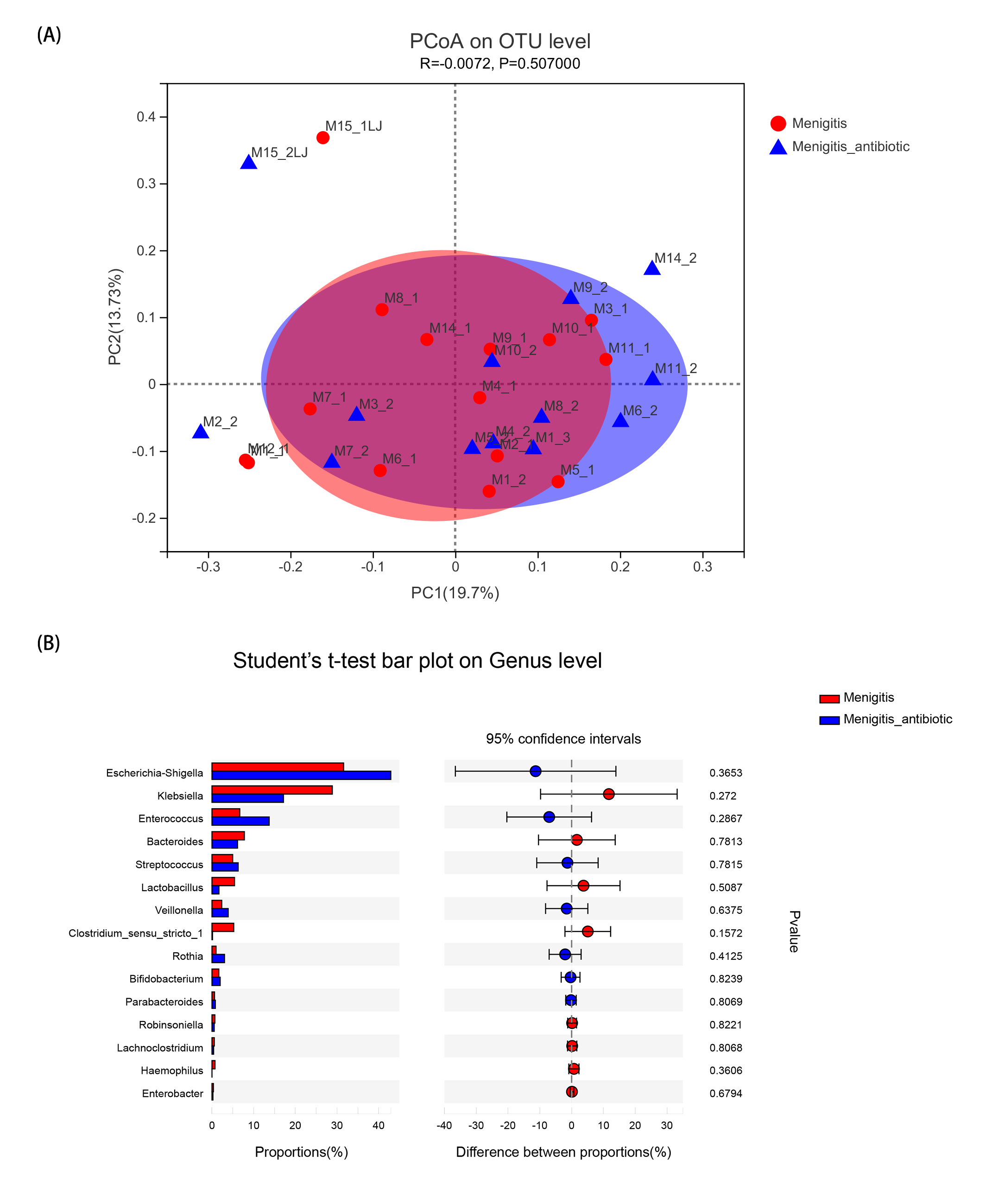

Supplement: Supplementary Figure 4 — Difference of gut microbiota before and after the use of antibiotics in meningitis group. (A) PCoA analysis before and after the use of antibiotics. (B) Relative abundance of significantly different genera before and after the use of antibiotics. [file Image_4.tif]
